# Ligand-Independent EGFR Activation by Anchorage-Stimulated Src Promotes Cancer Cell Proliferation and Cetuximab Resistance via ErbB3 Phosphorylation

**DOI:** 10.3390/cancers11101552

**Published:** 2019-10-14

**Authors:** Masami Nozaki, Hiroki Yasui, Yuichi Ohnishi

**Affiliations:** 1 Department of Cell Biology, Research Institute for Microbial Diseases, Osaka University, Suita, Osaka 565-0871, Japan; s65b40a.v8@gmail.com (H.Y.); onishi_yu_ku@msn.com (Y.O.); 2 Second Department of Oral and Maxillofacial Surgery, Osaka Dental University, Hirakata, Osaka 573-1121, Japan

**Keywords:** EGFR, ErbB3, Src, phosphorylation, TK inhibitor, cetuximab

## Abstract

Activation of the epidermal growth factor receptor (EGFR) pathway plays an important role in the progression of cancer and is associated with a poor prognosis in patients. The monoclonal antibody cetuximab, which displays EGFR extracellular domain-specific binding, has proven effective in the treatment of locally advanced disease and relapsed/metastatic disease. However, the effects of cetuximab are weaker than those of EGFR tyrosine kinase inhibitors (TKIs). This study investigates differences in the effects on cell growth of cetuximab and EGFR TKI AG1478 at the molecular level using oral squamous cell carcinoma (OSCC) cell lines. First, we found that there were EGFR-inhibitor-sensitive (EIS) and EGFR-inhibitor-resistant cell lines. The EIS cell lines expressed not only EGFR but also ErbB3, and both were clearly phosphorylated. The levels of phosphorylated ErbB3 were unaffected by cetuximab but were reduced by AG1478. EGFR ligand treatment increased the levels of phosphorylated EGFR but not phosphorylated ErbB3. Moreover, when EIS cell lines that were only capable of anchorage-dependent growth were grown in suspension, cell growth was suppressed and the levels of phosphorylated focal adhesion kinase (FAK), Src, and ErbB3 were significantly reduced. The levels of phosphorylated ErbB3 were unaffected by the FAK inhibitor PF573228, but were reduced by Src inhibition. Finally, combining cetuximab and a Src inhibitor produced an additive effect on the inhibition of EIS cell line growth.

## 1. Introduction

In 2018, approximately 355,000 people were diagnosed with, and 177,000 people died from, oral cancer worldwide, making it one of the most common human malignancies [1]. More than 90% of oral cancer is squamous cell carcinoma (SCC), and treatment usually involves surgical resection combined with chemotherapy and radiotherapy [2,3]. However, extensive surgery can lead to severe complications in advanced cancer patients. In addition, SCC treatment frequently involves platinum-based cytotoxic radiosensitizers, which may lead to poor prognoses and high mortality rates among oral SCC (OSCC) patients [4,5]. Therefore, new combination therapies are required for OSCC patients with recurrence and/or metastasis.

Epidermal growth factor receptor (EGFR) or ErbB1/human EGFR (HER) 1 is a receptor tyrosine kinase (RTK) and a member of the ErbB family (also known as the EGFR family or type I receptor family). Other members of this family include ErbB2/HER2/Neu, ErbB3/HER3, and ErbB4/HER4 [6,7]. The ErbB proteins share structural homologies, including a glycosylated extracellular domain that specifically binds to a ligand, a hydrophobic transmembrane domain, and a cytoplasmic domain with tyrosine kinase (TK) activity and phosphorylation sites [8]. Ligand binding to the extracellular domain alters the conformation of the receptor, resulting in homo- or hetero-dimerization and stimulating intrinsic TK activity. However, ErbB2 has no ligand binding site and ErbB3 lacks TK activity. Consequently, these proteins must form heterodimers to function effectively. Dimerization of ErbBs stimulates mutual autophosphorylation, and the signal is transmitted downstream by binding to molecules with Src homology 2 or pTyr-binding domains [6].

EGFR is highly expressed in most OSCCs and plays an important role in OSCC progression [9,10]. Therefore, EGFR may be useful as both a prognostic marker and a therapeutic target. In fact, the EGFR-specific antibody cetuximab has recently been combined with platinum-based chemotherapy in clinical practice to treat recurrent/metastatic OSCC [11]. Cetuximab is a recombinant chimeric monoclonal antibody that combines the Fv region of the mouse anti-EGFR antibody with the human immunoglobulin G1 (IgG1) heavy- and *κ* light-chain regions. Cetuximab specifically binds to the extracellular domain of EGFR and inhibits ligand–receptor binding, suppressing receptor dimerization and subsequent autophosphorylation. By blocking extracellular signal transduction, cetuximab can induce apoptosis and inhibit the cell cycle and angiogenesis, as well as cell migration [12,13].

Lapatinib, a dual TK inhibitor (TKI) that targets EGFR/ErbB2, has also proved effective in preclinical trials [14,15,16,17]. Lapatinib binds strongly but reversibly to the TK domains of both EGFR and ErbB2, thereby reducing the autophosphorylation of tyrosine residues. Because lapatinib inhibits ligand-induced signal transduction, its effects on EGFR are similar to those of cetuximab. However, when EGFR and ErbB2 are simultaneously overexpressed in patients with head and neck SCC, they form heterodimers and create intense proliferative signals [18]. Therefore, the dual inhibitor lapatinib may be more effective against tumors in general than cetuximab, which only acts on EGFR. We previously investigated the effects of lapatinib at the molecular level and observed that the levels of phosphorylated ErbB3 were reduced independently of those of EGFR and ErbB2 [19]. Furthermore, the EGFR TKI AG1478 inhibited the growth of OSCC cell lines more effectively than did cetuximab [20]. These results suggest that the EGFR-targeted anti-cancer effects of EGFR TKIs and cetuximab differ, and the difference in effect is linked to ErbB3 signaling.

In this study, we investigated differences in the anticancer effects of AG1478 and cetuximab at the molecular level using OSCC cell lines. The results show that EGFR signaling may stimulate growth by both ligand-dependent and -independent pathways, and that, while cetuximab only affects ligand-dependent growth, EGFR TKIs can suppress both pathways. Furthermore, we found that ligand-independent EGFR activation may be induced by anchorage-dependent Src activity, and that subsequent signaling, mediated by phosphorylation of ErbB3, leads to cell proliferation.

## 2. Results

### 2.1. AG1478 Suppresses Growth of Some Cancer Cell Lines More Effectively than Does Cetuximab, but Does not Alter the Growth of Cancer Stem-Like Cells

**Figure 1:**
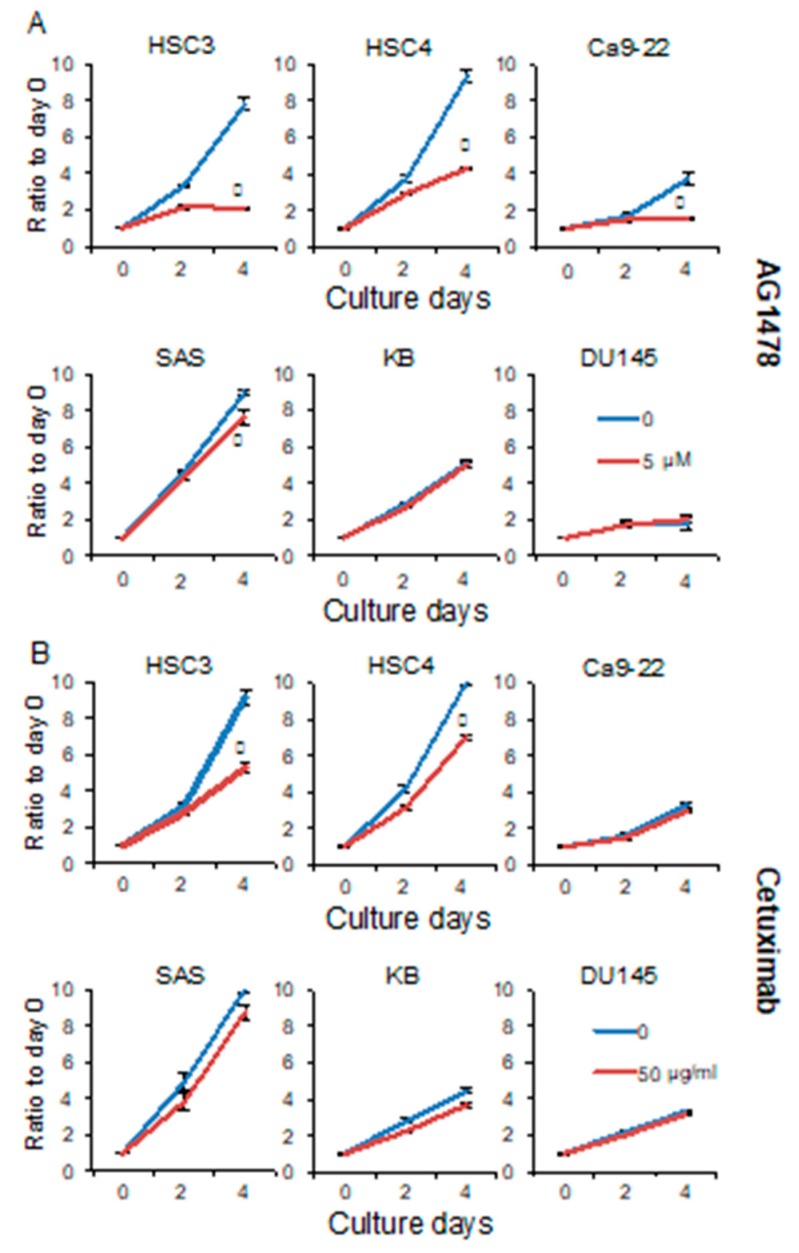
AG1478 and cetuximab have different inhibitory effects on growth of epidermal growth factor receptor (EGFR) inhibitor sensitive (EIS) cancer cells. The HSC3, HSC4, Ca9-22, SAS, KB, and DU145 cells were treated with (**A**) 5 μM of AG1478 or (**B**) 50 μg/mL of cetuximab, and MTS assays were performed on days 0, 2, and 4 to assess cell proliferation. A cetuximab of 10 μg/mL is sufficient to suppress cell growth. In this experiment, 50 μg/mL was used to reliability determine cetuximab resistance. Blue lines represent untreated control. Red lines represent AG1478 treatment on panel A and cetuximab treatment on panel B. * Significantly different from control, *p* < 0.05. Experiments were performed three times independently.

To investigate the role of EGFR in the proliferation of the OSCC cell lines HSC3, HSC4, Ca9-22, SAS, and KB, we performed 3-(4,5-dimethylthiazol-2-yl)-5-((3carboxymethoxyphenyl)-2-(4-sulfophenyl)-2-H-tetrazolium inner salt (MTS) assays after inhibitor treatment. The growth of HSC3, HSC4, and Ca9-22 cells was strongly inhibited by AG1478, which is an EGFR tyrosine kinase inhibitor (TKI). MTS assays also showed a significant decrease in the proliferation of SAS cells on day 4 of treatment, however, this inhibitory effect was weaker than that observed in the HSC3, HSC4, and Ca9-22 cell lines. The proliferation of KB cells was unaffected by AG1478 (Figure 1A). Next, we investigated the effect of cetuximab on the growth of OSCC cell lines. Cetuximab specifically binds to the extracellular domain of EGFR and inhibits ligand–receptor binding. MTS assays showed a significant decrease in the proliferation of HSC3 and HSC4 cells on day 4 of cetuximab treatment. The other cell lines grew as effectively in the presence of cetuximab as did untreated control cells (Figure 1B). These results show that the OSCC cell lines can be separated into EGFR-dependent and -independent proliferating groups. We also showed that there were significant differences in the sensitivities of the cells to the inhibitors. In addition, none of the AG1478-sensitive cell lines were capable of anchorage-independent growth and sphere formation [19]. In contrast, the SAS and KB cell lines, which had little or no sensitivity to AG1478 inhibition, displayed anchorage-independent growth and were able to form spheres. We investigated the EGFR-dependence of DU145, a prostate cancer cell line that could form spheres and found that it was as resistant to AG1478 and cetuximab as the KB cell line [19]. These data suggest that anchorage-dependent growth may be linked to EGFR dependence.

### 2.2. Epidermal Growth Factor Receptor (EGFR) TKI Inhibits the Phosphorylation of Both EGFR and ErbB3

**Figure 2:**
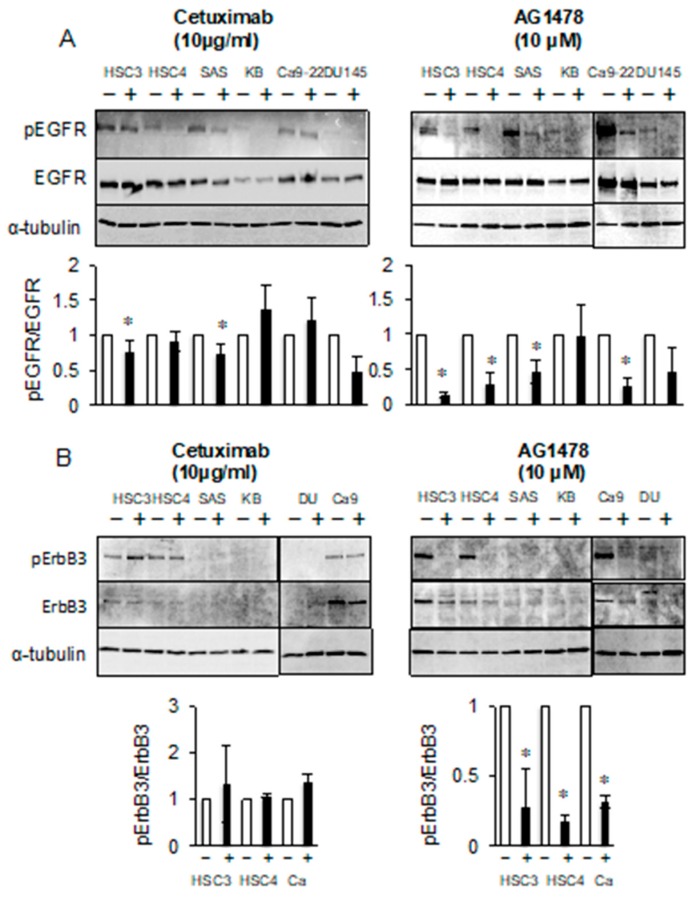
AG1478 and cetuximab have different inhibitory effects on ErbB signaling of cancer cells. The HSC3, HSC4, Ca9-22, SAS, KB, and DU145 cells were treated with 10 μg/mL of cetuximab or 10 μM of AG1478 and the phosphorylation and protein levels of (**A**) epidermal growth factor receptor (EGFR) and (**B**) ErbB3 were determined by western blotting analysis. *α*-tubulin was used as the loading control. * Significantly different from control, *p* < 0.05. Experiments were performed three times independently.

Differences in sensitivity to EGFR inhibitors may be due to differences in EGFR protein levels. However, EGFR protein expression was clearly detected in all of the cell lines that were investigated. In addition, EGFR expression levels were similar in both the presence and absence of inhibitors (Figure 2A). These results indicate that the differences in sensitivity to EGFR inhibitors that are reflected in cell growth do not depend on the expression levels of EGFR protein.

Next, differences in the effects of AG1478 and cetuximab treatment on EGFR phosphorylation were investigated by western blotting analysis using a specific antibody (Figure 2A). The levels of phosphorylated EGFR were significantly reduced by AG1478 treatment in all cells without KB and DU145 cells. The levels of phosphorylated EGFR in the EGFR-inhibitor resistant (EIR) KB and DU145 cell lines were low before treatment with AG1478 and nearly absent following treatment. Reduced levels of phosphorylated EGFR were observed in the HSC3 and SAS cell lines after cetuximab treatment, and the reduced levels of phosphorylated EGFR observed in the HSC4 and Ca9-22 cell lines were not remarkable by comparison (Figure 2A).

EGFR is one of four ErbB family members, and it can function as a homodimer or as a heterodimer with other ErbBs. In the case of heterodimeric receptors, the phosphorylation of the other ErbB family members may be suppressed by EGFR inhibitors. We previously showed that all four ErbB family members were expressed in OSCC cells and that ErbB3 was expressed in HSC3, HSC4, and Ca9-22 cell lines. Furthermore, we reported that the EGFR/ErbB2 dual inhibitor lapatinib suppresses the phosphorylation of ErbB3 [19]. Phosphorylated ErbB3 was almost absent after AG1478 treatment. However, ErbB3 phosphorylation was not significantly affected by cetuximab treatment (Figure 2B). Because ErbB3 has no cytoplasmic TK activity [8], these results suggest that ErbB3 can form a heterodimer with EGFR and is phosphorylated by ligand-independent EGFR TK activity.

**Figure 3:**
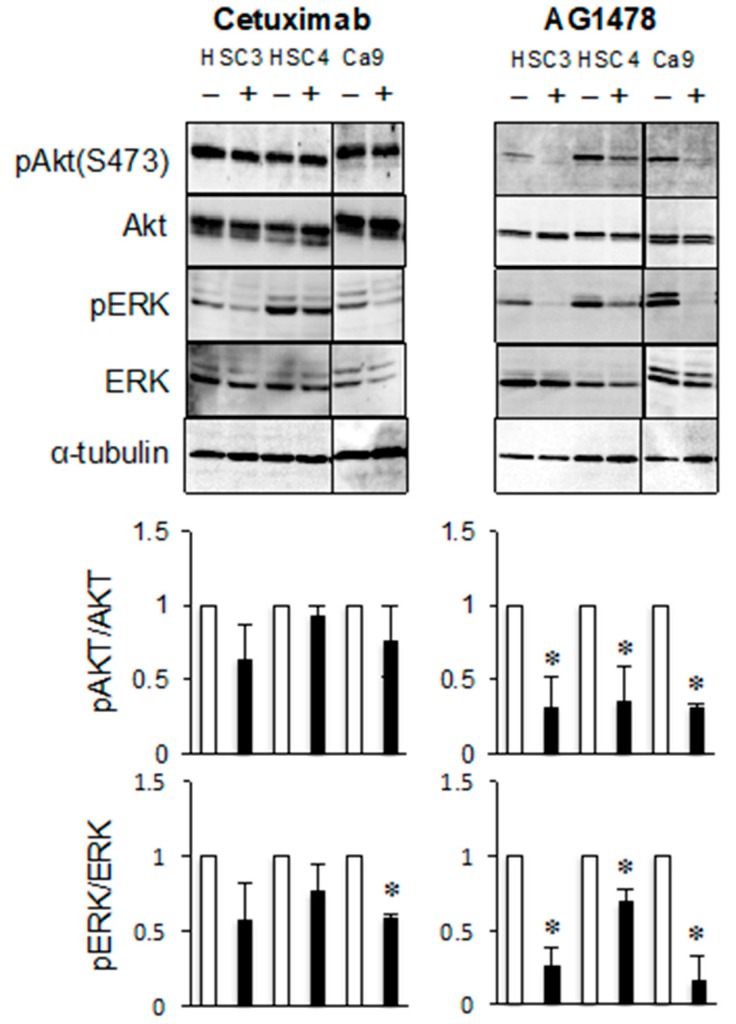
AG1478 and cetuximab have different inhibitory effects on ErbB3 signaling pathway of cancer cells. The HSC3, HSC4, and Ca9-22 cells were treated with cetuximab or AG1478 and the phosphorylation and protein levels of AKT and extracellular signal-related kinase (ERK) were determined by western blotting analysis. *α*-tubulin was used as the loading control. * Significantly different from control, *p* < 0.05. Experiments were performed three times independently.

The effects of AG1478 on the phosphorylation of AKT and extracellular signal-related kinase (ERK) were investigated, because these proteins may be downstream signaling components of the EGFR–ErbB3 pathway. The levels of phosphorylated AKT and phosphorylated ERK were clearly reduced after AG1478 treatment. However, cetuximab treatment decreased the levels of phosphorylated ERK in Ca9-22 cells but had little effect on the levels of phosphorylated AKT (Figure 3). These results indicate that ligand-dependent EGFR activity and ligand-independent EGFR–ErbB3 activity regulate different signal transduction pathways.

### 2.3. Phosphorylation of ErbB3 is not due to Ligand-Dependent EGFR TK Activity

**Figure 4:**
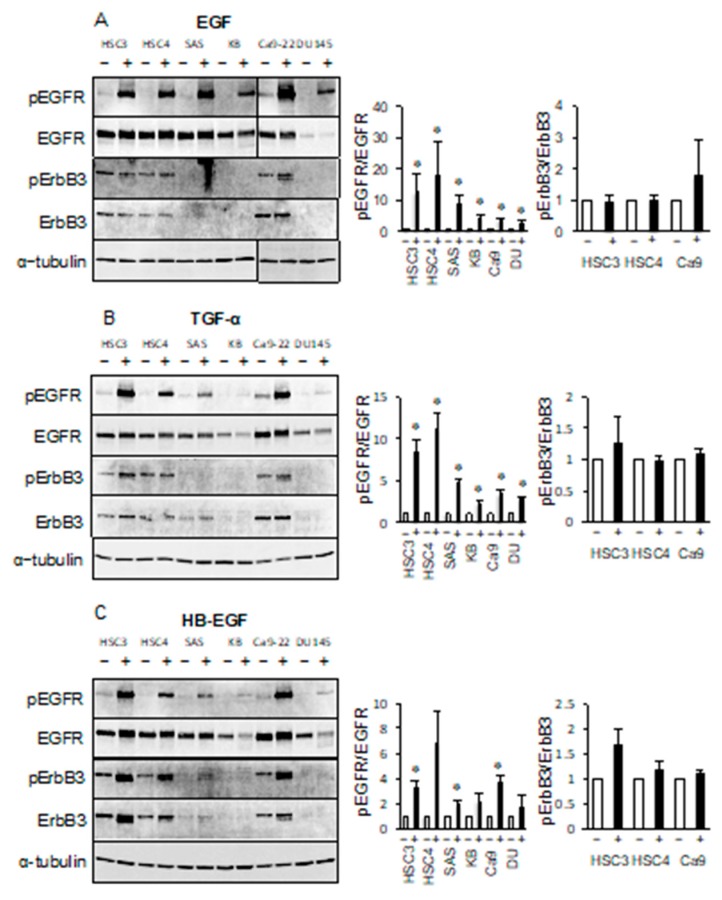
EGFR ligands are not involved in the phosphorylation of ErbB3. Cells were treated with (**A**) epidermal growth factor (EGF), (**B**) transforming growth factor-*α* (TGF-*α*), (**C**) heparin-binding EGF-like growth factor (HB-EGF), and the phosphorylation levels of EGFR and ErbB3 were determined by western blotting analysis. *α*-tubulin was used as the loading control. * Significantly different from control, *p* < 0.05. Experiments were performed three times independently.

To show that EGFR ligands do not affect the phosphorylation of ErbB3, we investigated the phosphorylation of EGFR and ErbB3 in cancer cell lines treated with EGF, transforming growth factor-*α* (TGF-*α*), or heparin-binding EGF-like growth factor (HB-EGF). We confirmed that the levels of phosphorylated EGFR were increased by EGF, TGF-*α*, or HB-EGF treatment in all cell lines (Figure 4). In contrast, EGF treatment produced almost no change in the levels of phosphorylated ErbB3 in the HSC3, HSC4, or Ca9-22 cell lines (Figure 4A). TGF-*α* treatment did produce a slight increase in the levels of phosphorylated ErbB3 in HSC3 cells but no significant changes in the HSC4 or Ca9-22 cell lines (Figure 4B). Neither EGF nor TGF-*α* treatment produced any increase in the levels of phosphorylated ErbB3 in the SAS, KB, or DU145 cell lines. The levels of phosphorylated ErbB3 were increased by HB-EGF treatment, compared to untreated cells. However, the levels of ErbB3 protein were also increased by HB-EGF treatment (Figure 4C). Therefore, this increase in the levels of phosphorylated ErbB3 levels appears to be due to an increase in the quantity of protein and not an increase in phosphorylation levels. These results suggest that the phosphorylation of ErbB3 in the HSC3, HSC4, and Ca9-22 cell lines was not due to ligand-stimulated EGFR activity.

**Figure 5:**
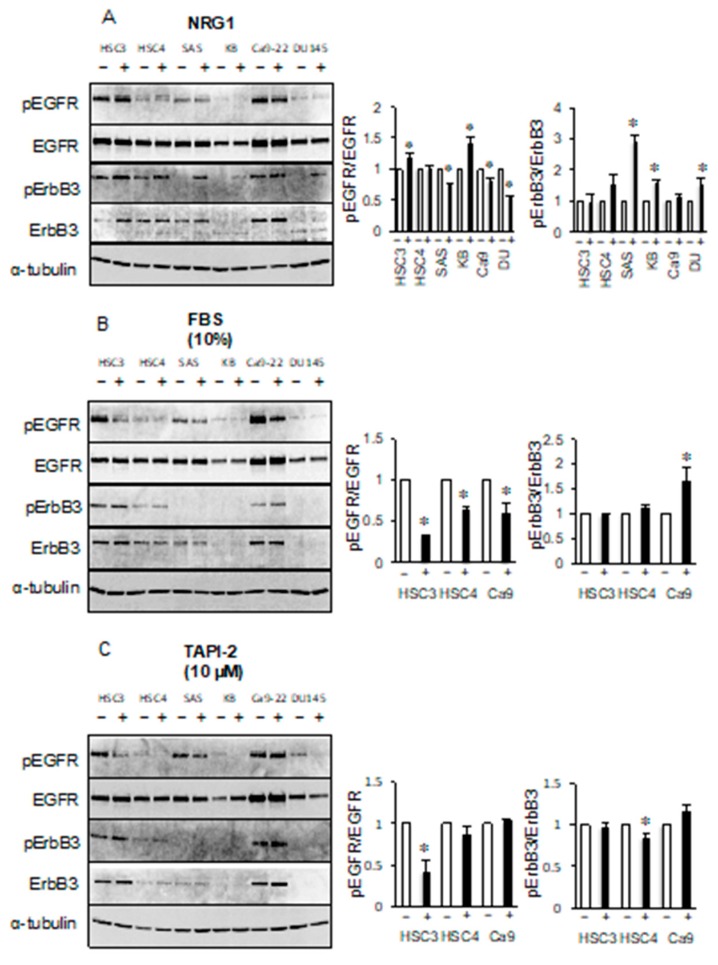
Extracellular signals are not involved in the phosphorylation of ErbB3. Cells were treated with (**A**) neuregulin 1 (NRG1), (**B**) 10% fetal bovine serum (FBS), and (**C**) 10 μM tumor necrosis factor alpha protease inhibitor 2 (TAPI-2) and the phosphorylation levels of EGFR and ErbB3 were determined by western blotting analysis. *α*-tubulin was used as the loading control. * Significantly different from control, *p* < 0.05. Experiments were performed three times independently.

The ErbB3 ligand neuregulin 1 (NRG1) increased the levels of phosphorylated EGFR in HSC3 and KB cell lines and decreased the levels of EGFR in SAS, Ca9-22, and DU145 cell lines. NRG1 increased the levels of phosphorylated ErbB3 in the SAS, KB, and DU145 cell lines, but had little effect in the HSC3, HSC4, and Ca9-22 cell lines (Figure 5A). Binding of ErbB3 to NRG1 in the SAS, KB, or DU145 cell lines promotes heterodimer formation involving ErbB2 or ErbB4, suggesting that the levels of phosphorylated ErbB3 may increase. Heterodimerization with EGFR in the absence of a ligand may be mainly responsible for the phosphorylation of ErbB3 in HSC3, HSC4, and Ca9-22 cells.

Next, we investigated whether EGFR/ErbB3 could be activated by unknown serum factors. Interestingly, the addition of fetal bovine serum (FBS) in serum-free culture reduced the levels of phosphorylated EGFR in the HSC3, HSC4, and Ca9-22 cell lines. However, the levels of phosphorylated ErbB3 appeared to be unchanged in HSC3 and HSC4 cell lines, but increased in Ca9-22 cell line by the addition of FBS (Figure 5B). We also investigated whether factors released by shedding had a role in these pathways by performing treatments with the sheddase inhibitor TAPI-2. We observed a decrease in the levels of phosphorylated EGFR in HSC3 cell line by TAPI-2 treatment but no change in the phosphorylated ErbB3 in HSC3. ErbB3 phosphorylation level of HSC4 was decreased by TAPI-2 treatment, but the EGFR phosphorylation level was not affected (Figure 5C). These results indicate that soluble factor(s), including autocrine factors, which can regulate EGFR activity, do not appear to alter the levels of phosphorylated ErbB3.

### 2.4. EGFR/ErbB3 Signal Transduction is Associated with Anchorage-Dependent Cell Growth

**Figure 6:**
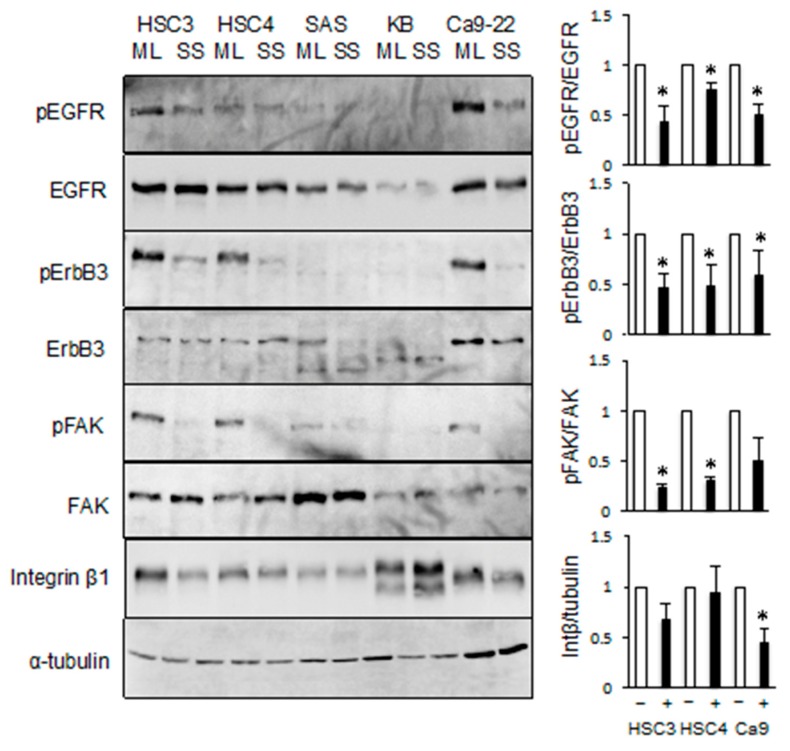
The phosphorylation levels of focal adhesion kinase (FAK), EGFR, and ErbB3 were reduced in suspension cultures. HSC3, HSC4, SAS, KB, and Ca9-22 cells were cultured on ultra-low adhesion plates (suspension; SS) or tissue culture plates (monolayer; ML). The levels of phosphorylated EGFR, ErbB3, FAK, and integrin β1 were determined by western blotting analysis. α-tubulin was used as the loading control. * Significantly different from control, *p* < 0.05. Experiments were performed three times independently.

If EGFR stimulates ligand independent phosphorylation of ErbB3, what are the factors that induce EGFR activation? EGFR/ErbB3 stimulates anchorage-dependent proliferation of HSC3, HSC4, and Ca9-22 cells because these cells do not grow under suspension culture conditions [19]. Therefore, we investigated the phosphorylation of EGFR and ErbB3 in suspension culture. We found that growing these cells in floating culture in non-adhesion culture dishes induced dephosphorylation of EGFR and ErbB3 (Figure 6).

Epithelial cells undergo growth arrest when detached from the extracellular matrix. Focal adhesion kinase (FAK) integrins, and their downstream signaling components, have central roles in cell adhesion-mediated signal transduction [21,22]. The levels of phosphorylated FAK were markedly reduced when HSC3, HSC4, SAS, and Ca9-22 cells were grown in suspension. The levels of integrin *β*1 were slightly reduced (Figure 6).

### 2.5. Phosphorylation of ErbB3 is Stimulated by Src but Not Focal Adhesion Kinase (FAK)

**Figure 7:**
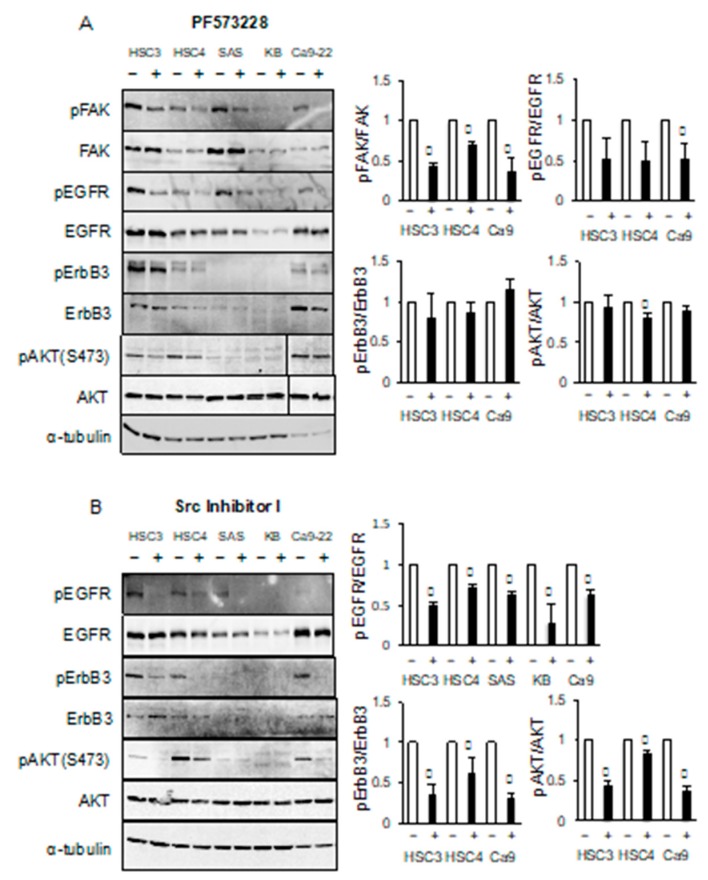
Src inhibitor reduces the phosphorylation levels of EGFR, ErbB3, and AKT. HSC3, HSC4, SAS, KB and Ca9-22 cells were treated (**A**) with or without PF573228 or (**B**) Src inhibitor-1. The levels of phosphorylated FAK (pFAK), FAK, phosphorylated EGFR (pEGFR), EGFR, phosphorylated ErbB3 (pErbB3), ErbB3, phosphorylated AKT (pAKT), and AKT were determined by western blotting analysis. *α*-tubulin was used as the loading control. * Significantly different from control, *p* < 0.05. Experiments were performed at least three times independently.

To investigate whether the reductions in the levels of phosphorylated FAK that occurred when cells were grown in suspension culture were associated with EGFR-mediated ErbB3 phosphorylation, the levels of phosphorylated EGFR and ErbB3 in cells treated with the FAK-specific inhibitor, PF573228, were determined by western blotting analysis. The levels of phosphorylated EGFR in HSC3, HSC4, SAS, and Ca9-22 cells were decreased by treatment with PF573228. However, there was little difference in the levels of phosphorylated ErbB3 between treated and untreated cells (Figure 7A). Furthermore, PF573228 treatment had a weak effect on AKT phosphorylation (Figure 7A) and proliferation (data not shown). These results suggest that FAK-mediated signaling may be unassociated with the EGFR/ErbB3/cell growth pathway.

Previous research has shown that G-protein-coupled receptors (GPCRs) and Src are involved in ligand-independent EGFR transactivation [23,24]. In this study, we investigated the role of Src in ligand-independent EGFR activation. Treatment with Src inhibitor-1 significantly reduced the levels of phosphorylated EGFR, and the levels of phosphorylated ErbB3 also decreased (Figure 7B). These results suggest that the EGFR–ErbB3 signaling pathway may be induced by Src.

**Figure 8:**
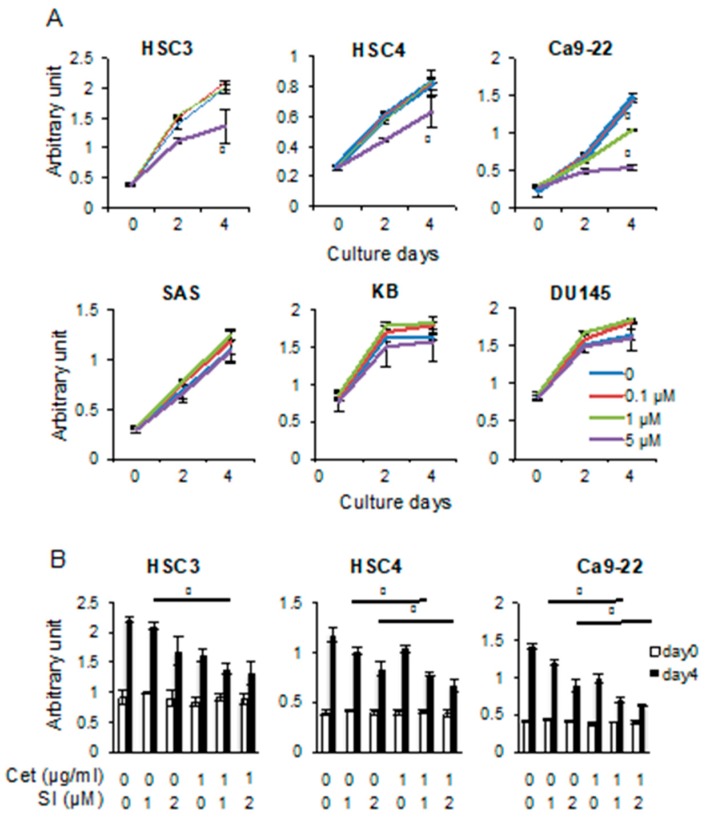
Src inhibitor suppresses cell growth of EIS cell lines more effectively in combination with cetuximab. (**A**) Src inhibitor-1 suppresses the growth of HSC3, HSC4, and Ca9-22 cells in concentration-dependent manner, but does not suppress the growth of SAS, KB, and DU145 cells. Treatment with 0 (blue), 0.1 μM (red), 1 μM (green), or 5 μM (purple) of Src inhibitor-1 was added and MTS assay was performed after 0, 2, and 4 days. (**B**) The combination of Src inhibitor-1 (SI) and cetuximab (cet) is more effective than each alone. After adding cet and SI at each concentration, MTS assay was performed 4 days later and compared with day 0. * Significantly different from control, *p* < 0.05. Experiments were performed three times independently.

We investigated the effect of Src-inhibitor treatment on the proliferation of cancer cells. We found that inhibiting Src had no effect on SAS, KB, and DU145 cells. However, treatment with 5 μM Src inhibitor-1 significantly reduced the proliferation of HSC3 and HSC4 cells. In addition, the proliferation of Ca9-22 cells was already significantly reduced with 1 μM of Src inhibitor-1 and further decreased with 5 μM of Src inhibitor-1 (Figure 8A). Furthermore, combining Src inhibitor-1 with cetuximab suppressed cell proliferation more effectively than did Src-inhibitor treatment alone (Figure 8B). These data suggest that the proliferation of these cancer cells was affected by a combination of ligand-dependent and -independent EGFR activation.

## 3. Discussion

**Figure 9:**
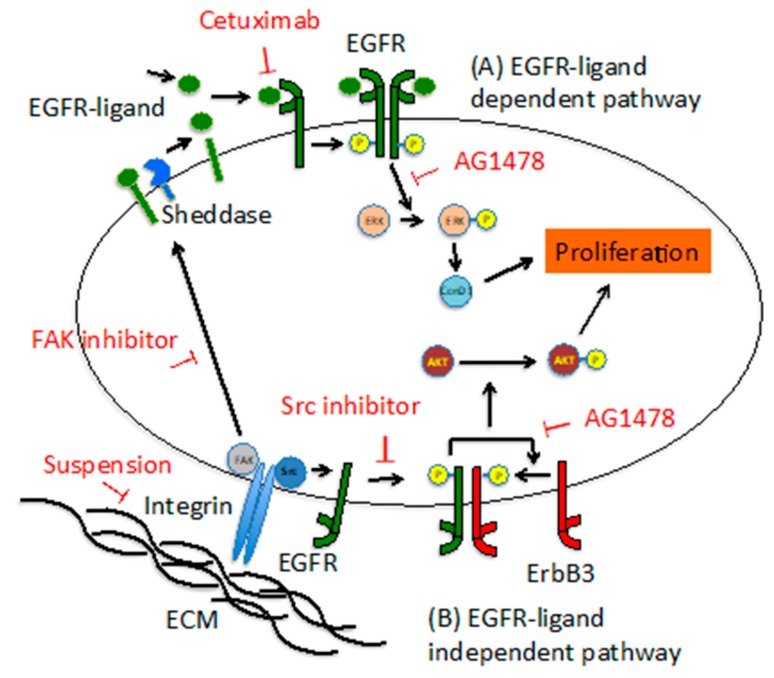
Models of EGFR-ligand dependent pathway and EGFR-ligand independent pathway in EIS cells. (**A**) In the EGFR-ligand dependent pathway, proliferation is promoted via ERK/cyclin D1 by EGFR activated by the binding of an EGFR-ligand and EGFR. The activity of FAK or sheddase may be involved in EGFR phosphorylation. (**B**) In the EGFR-ligand independent pathway, EGFR transactivated via Src by cell-substratum adhesion phosphorylates ErbB3 and promotes proliferation via AKT.

In this study, we observed that the OSCC cell lines included EGFR-inhibitor sensitive (EIS; HSC3, HSC4, and Ca9-22) and EIR (SAS, KB, and DU145) cell lines. The growth of EIS cell lines was anchorage-dependent and regulated by ligand-dependent and -independent EGFR pathways. Because cetuximab only inhibited the ligand-dependent EGFR pathway, its inhibitory effects were weaker than those of EGFR TKIs, which inhibited both pathways. Furthermore, the phosphorylation of ErbB3 by a ligand-independent EGFR pathway that was transactivated via Src in response to cell-substratum adhesion was apparently important for cell proliferation. In addition, the combination of cetuximab and a Src inhibitor may provide more effective OSCC therapy than either inhibitor alone. A summary of the results and a model diagram are shown in Figure 9.

Previous research has highlighted the importance of ErbB3 in cancer progression. For many tumors, the overexpression of ErbB3 is a poor prognostic factor and is associated with low patient survival rates [25,26,27,28,29]. ErbB3 is involved in the development of anti-EGFR and anti-ErbB2 therapy resistance [30,31,32]. In addition, because ErbB3 has an intracellular domain containing multiple tyrosine phosphorylation sites that recruit phosphatidylinositol 3 kinase (PI3K) regulatory p85 subunits, it can activate the PI3K/AKT pathway more effectively than other ErbB family proteins [33]. Most of the previous research investigating the importance of ErbB3 has focused on the ErbB2/ErbB3 heterodimer [33,34]. However, treatment with AG1478 decreased the levels of phosphorylated AKT more effectively than did treatment with cetuximab, in HSC3 and Ca9-22 cells (Figure 3). This suggests that the EGFR/ErbB3 heterodimer also plays an important role in signal transduction. A transition from EGFR-homodimer to EGFR/ErbB3-heterodimer signaling in response to persistent treatment with EGFR inhibitors can reactivate the PI3K/AKT pathway and evade the therapeutic action of these inhibitors [35]. Therefore, this study showed how EGFR that is transactivated by a signal within the cytoplasm may activate the ErbB3-mediated signaling pathway in cancer cells with acquired resistance to cetuximab treatment.

Treatment with the ErbB3-ligand NRG1 demonstrated that the levels of phosphorylated ErbB3 had little effect on EIS cells. However, while no phosphorylation was detected in untreated EIR cells, ErbB3 was clearly phosphorylated in response to treatment with NRG1 (Figure 5A). Therefore, like EGFR, ErbB3 may also stimulate signaling responses in the absence of a ligand in EIS cells, although the possibility that NRG2 acts as an ErbB3 ligand cannot be discounted [36]. According to the EGFR/ErbB3 rotation model, EGFR and ErbB3 bind to their respective ligands after heterodimerization [37,38]. Therefore, in cells that require an EGFR/ErbB3 signal for growth, EGFR that is transactivated before or after the formation of a heterodimer may phosphorylate ErbB3 and transmit a specific signal. Furthermore, ErbB3, which appeared to be inactive in EIR cells, became functional in response to artificial ligand stimulation. EGFR ligands did not affect the levels of phosphorylated ErbB3, suggesting that ErbB3 forms heterodimers with ErbB2 or ErbB4, and may become phosphorylated and functional in EIR cells. Therefore, the function of cells may depend on their environment. For example, a study of the activity of ErbB3 demonstrated the importance of the tumor microenvironment [39].

This study showed that cell lines that are only capable of anchorage-dependent growth expressed high levels of phosphorylated ErbB3. Patients with head and neck SCC who have also been infected with human papillomavirus (HPV) may show high levels of phosphorylated ErbB3 expression, and ErbB3 may be a therapeutic target [40]. However, HPV morbidity in patients with OSCC is only approximately 7.0% [41], and the OSCC cell lines used in previous studies may not be HPV-positive [42,43]. Nevertheless, some cells are HPV-negative but have high ErbB3 levels [40], and the ErbB3-positive rate is extremely high in OSCC patients [44]. Therefore, we decided that it was unnecessary to consider the relationship between ErbB3 levels and HPV infection in OSCC patients.

The phosphorylation of ErbB3 was unaffected by cetuximab but nearly absent following treatment with AG1478. Because ErbB3 has little TK activity, it may be trans-phosphorylated by the ErbB protein that completes the heterodimer [45]. Therefore, the ErbB3 phosphorylation observed in this study is apparently induced by ligand-independent EGFR activity, which is not affected by cetuximab. If there are no suitable ligands and no TK activity in the ErbB3-dimerization pair, another molecule is required for EGFR activation and phosphorylation. EGFR has an extracellular domain and an intracellular kinase domain with a long regulatory C-terminal tail. These domains are linked by a single-helix transmembrane segment and a juxta-membrane segment. Usually, ligand binding and dimerization stimulates EGFR kinase activity. This kinase activity trans-phosphorylates the tyrosine residues of the C-terminal tail of the dimerization partner [46,47,48]. A functional analysis of each domain demonstrated that, although the EGFR TK domain with the juxta-membrane segment is strongly activated by forming a dimer [49], if the full-length EGFR is localized on the cell membrane, then the TK activity is only stimulated in the presence of a ligand [50]. Even the isolated extracellular domains are capable of forming dimers [51]. However, although EGFR has intrinsic TK activity, this is auto-inhibited by the conformation adopted by the TK domain in the absence of ligand binding, even if EGFR is dimerized on the membrane [51]. This study suggests that Src, downstream of the anchorage signal, may be associated with EGFR phosphorylation. Therefore, the ligand-independent EGFR activity observed may be due to conformational changes in the EGFR cytoplasmic domain and activation of TK activity in response to an anchorage-induced Src signal.

The ErbB network can integrate various signals including hormones, neurotransmitters, lymphokines and stress inducers, as well as ligands [52]. These trans-regulatory interactions are mediated by protein kinases that phosphorylate and activate ErbBs [52]. The most widely studied mechanism involved in integrating these networks is the GPCR pathway. Interestingly, the mitogenic activity of some GPCR agonists requires transactivation of ErbBs. These agonists induce the phosphorylation of EGFR by stimulating its autophosphorylation [53]. A Src kinase recruited by calcium-regulated proline-rich TK-2 [54] or a GPCR-coupled kinase and adaptor protein [55] is also involved [56].

Phosphorylation of EGFR tyrosine 845 (Y845) is also important for EGFR TK activity [57,58]. TGF-*β* stimulates the phosphorylation of EGFR Y845, via Src activation, as part of a ligand-independent pathway [24]. In addition, ozone trans-phosphorylates EGFR Y845 and Y1068 via Src activation in human branchial epithelial cells [59]. Therefore, ligand-independent EGFR transactivation may also lead to intrinsic TK activation in response to a Src signal induced by anchorage.

SAS cell line was classified as EIR, but on the fourth day of 5 μM of AG1478 treatment, it appeared that the growth rate was reduced compared to untreated control (Figure 1A). These data suggest that EGFR may be involved, albeit weakly, in SAS proliferation. So far, the SAS cell line has not been sensitive to 10 μg/mL of cetuximab [60], but it cannot be denied the sensitivity to higher concentration of cetuximab. Therefore, in this study, MTS assay was performed with 50 μg/mL of cetuximab. The results showed that the SAS cell line, together with KB and DU145 cell lines, is resistant to high concentrations of cetuximab. The EIR cell lines were capable of anchorage-independent growth and could form spheres. These characteristics are typical of cancer stem cells [61] and may originate in small populations of cells that are involved in the initiation, metastasis, and recurrence of cancer. However, more than 80% of OSCCs are ErbB3-positive [44]. Therefore, most cancer tissues probably contain anchorage-dependent ErbB3 positive cells. We previously suggested that cetuximab treatment may reduce cell migration in some OSCC cell lines [60,62,63], thereby inhibiting metastasis. We believe that combining cetuximab and Src inhibitors will lead to effective therapeutic strategies that prevent tumor progression.

## 4. Materials and Methods

### 4.1. Cell Culture and Reagents

The OSCC cell lines HSC3, HSC4, SAS, Ca9-22, and KB, and the prostate cancer cell line DU145 used in this study were purchased from the RIKEN BioResource Center (Ibaraki, Japan). Cells were cultured in Dulbecco’s modified Eagle’s medium (DMEM) supplemented with 10% (v/v) fetal bovine serum (FBS) at 37 °C in a humidified atmosphere containing 5% CO_2_. DMEM and FBS were purchased from Gibco (Life Technologies, Tokyo, Japan). Cetuximab (Erbitux) was purchased from Merck Serono, Co., Ltd. (Tokyo, Japan). AG1478 and tumor necrosis factor protease inhibitor 2 (TAPI-2) were from Calbiochem (Merk Millipore, Tokyo, Japan), Src inhibitor-1 and PF573228 were from Sigma-Aldrich (Tokyo, Japan). Transforming growth factor (TGF)-*α* and neuregulin (NRG) 1 were purchased from R and D Systems (Minneapolis, MN, USA). HB-epidermal growth factor (EGF) was purchased from Wako Pure Chemical Industries, Ltd. (Osaka, Japan).

### 4.2. Cell Proliferation Assay

Cancer cells (1 × 10^3^/well) were seeded into 96-well plates. After 24 h of growth, cetuximab, AG1478, or Src inhibitor-1 were added at the concentrations indicated, and the cells were allowed to grow for 1 additional day. Cell proliferation was assessed using the CellTiter 96^®^ Aqueous One Solution Cell Proliferation MTS assay kit (Promega, Tokyo, Japan). Assays were performed in accordance with the manufacturer’s instructions. It has been confirmed that the decrease in MTS values due to cetuximab and AG1478 correlates with the decrease in cell number (Supplementary Figure S1).

### 4.3. Floating Culture

For suspension culture, separated cells were plated into wells of ultra-low attachment 6-well plates and cultured in DMEM supplemented with 10% (v/v) FBS at 37 °C under 5% CO_2_.

### 4.4. Western Blotting Analysis

Cells were washed with phosphate-buffered saline (PBS) and then lysed in radioimmunoprecipitation assay buffer (10 mM Tris-HCl, pH 8.0; 150 mM NaCl; 1% Nonidet P-40; 0.1% sodium dodecyl sulfate (SDS); 0.5% sodium deoxycholate; and 5 mM ethylenediaminetetraacetic acid (EDTA)) supplemented with 1 × Halt^TM^ Protease inhibitor Cocktail and 1 × Halt^TM^ Phosphate inhibitor Cocktail (both from Thermo Fisher Scientific, Waltham, MA, USA). The protein concentrations of the lysates were determined using the BCA^TM^ Protein Assay Kit (Thermo Fisher Scientific), and equal amounts of the denatured proteins were separated using SDS–polyacrylamide gel electrophoresis. The separated proteins were transferred onto Clear Trans polyvinylidene difluoride membranes (Wako Pure Chemical Industries, Ltd.). After blocking with 5% skimmed milk in TBST buffer (20 mM Tris-HCl, pH 7.5; 150 mM NaCl; 0.05% Tween 20), the membranes were probed using the primary antibodies indicated, followed by washing in TBST and incubation with horseradish peroxidase-conjugated secondary antibodies (ECL^TM^ anti-mouse IgG, horseradish and ECL^TM^ anti-rabbit IgG; horseradish from GE Healthcare, Little Chalfont, UK). Protein bands were detected using ECL-Plus western blotting detection reagent (GE Healthcare).

### 4.5. Antibodies

The antibodies used were obtained from the following suppliers: EGFR, EGFR-pY1068, ErbB3, ErbB3-pY1289, AKT, AKT-pS473, focal adhesion kinase (FAK), and FAK-pY397 were from Cell Signaling Technology (Danvers, MA, USA), Extracellular signal-related kinase (ERK) 1, ERK-pY204, and Integrin *β*1 were from Santa Cruz Biotechnology (Dallas, TX, USA), and *α*-tubulin was from Sigma-Aldrich.

### 4.6. Statistical Analyses

Statistical analyses were performed using one-way analysis of variance (ANOVA). Statistical significance (* *p* < 0.05) was evaluated using unpaired Student’s *t*-tests to assess the differences between the treated and control samples.

## 5. Conclusions

In this study, we found that cell-substratum adhesion transactivated EGFR via Src signaling in some cancer cells. In addition, activated EGFR promoted growth by phosphorylating ErbB3. Therefore, we demonstrated the importance of the EGFR/ErbB3 pathway in the absence of a ligand. These cancer cells were non-cancer stem cells that did not form spheres and are considered mainly responsible for tumor expansion. In addition, because these cells also had ligand-stimulated EGFR activity, we were able to show that combining cetuximab with a Src inhibitor may enhance the therapeutic effect.

## Supplementary Materials

The following is available online at https://www.mdpi.com/2072-6694/11/10/1552/s1, Figure S1: MTS assay data for cetuximab and AG1478 treated cells reflect cell numbers.
