# Supplementary figures and images for: Ligand-Independent EGFR Activation by Anchorage-Stimulated Src Promotes Cancer Cell Proliferation and Cetuximab Resistance via ErbB3 Phosphorylation
Source: Cancers (Basel). 2019 Oct 14;11(10):1552. doi: 10.3390/cancers11101552 (PMC6826992; doi:10.3390/cancers11101552)

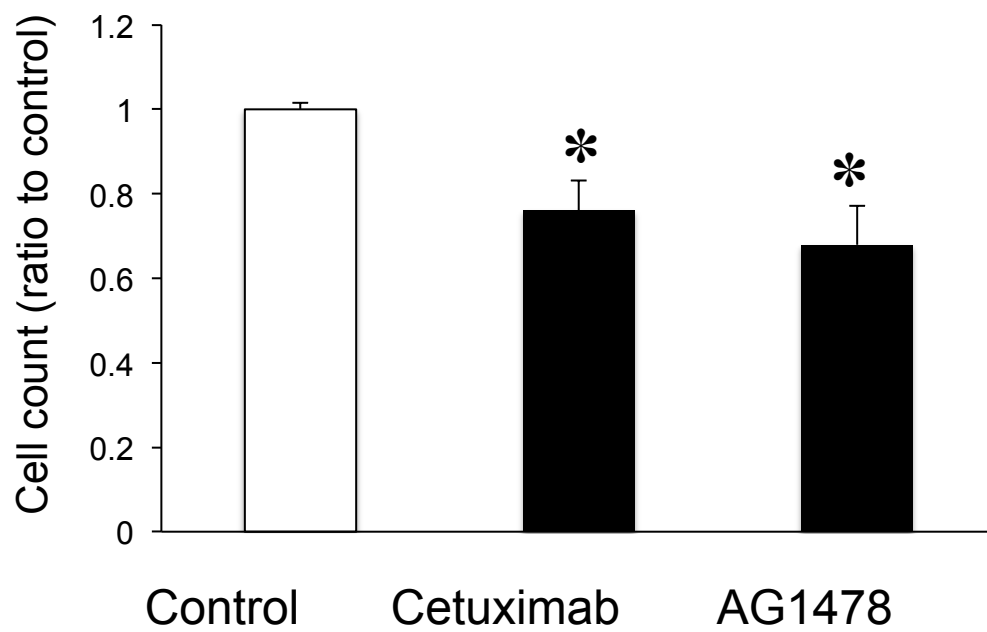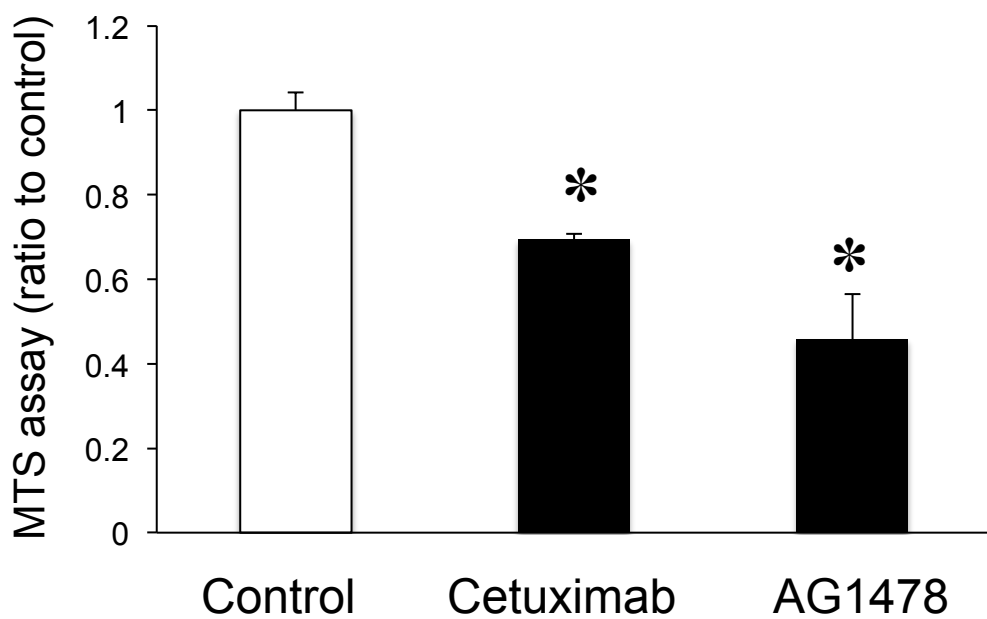

Supplement: Supplementary file 1 [file cancers-11-01552-s001.pdf]
